# PHYSICIANS LIVING WITH DEMENTIA CHAMPION CHANGE, PROVIDE HOPE AND REFUTE STIGMA

**DOI:** 10.1093/geroni/igac059.214

**Published:** 2022-12-20

**Authors:** Arnold Beresh, Brenda Roberts, Lisa Dedden Cooper

**Affiliations:** National Council of Dementia Minds, White Lake, Michigan, United States; National Council of Dementia Minds, Elwell, Michigan, United States; AARP Michigan, Owosso, Michigan, United States

## Abstract

This section of the symposium will show how to empower behavior change amongst the medical profession and the people they serve. Health care professionals who were diagnosing and treating dementia patients who are now themselves living with dementia will share insights into what needs to change by sharing “What I Wish I Knew Then.” We have asked three people from the National Council of Dementia Minds to join us for the symposium. Dr. Arnold Beresh, Brenda Roberts, and Lisa Cooper to speak on What I Wish I Knew Then. This part of the presentation is designed to provoke discussion on how best to engage doctors to support better brain health for older adults in the future.

